# Ethical dilemmas and the reconstruction of subjectivity in digital mourning in the age of AI: an empirical study on the acceptance intentions of bereaved family members of cancer patients

**DOI:** 10.3389/fdgth.2025.1618169

**Published:** 2025-07-07

**Authors:** Kun Fu, Chenxi Ye, Zeyu Wang, Miaohui Wu, Zhen Liu, Yuan Yuan

**Affiliations:** ^1^ School of Culture and Creativity, Beijing Normal-Hong Kong Baptist University, Zhuhai, Guangdong, China; ^2^ Faculty of Philosophy, Zhengzhou University, Zhengzhou, Henan, China; ^3^ School of Digital Art and Design, Chengdu Neusoft University, Chengdu, Sichuan, China

**Keywords:** AI digital mourning, UTAUT model, ethical concerns in AI, perception of grief, emprical study

## Abstract

**Introduction:**

With the rapid advancement of AI replication, virtual memorials, and affective computing technologies, digital mourning has emerged as a prevalent mode of psychological reconstruction for families coping with the loss of terminally ill patients. For family members of cancer patients, who often shoulder prolonged caregiving and complex ethical decisions, this process entails not only emotional trauma but also profound ethical dilemmas.

**Methods:**

This study adopts the Unified Theory of Acceptance and Use of Technology (UTAUT) as its analytical framework, further integrating Foucauldian subjectivation theory and emotional-cognitive models. A structural path model was constructed to examine how ethical identification and grief perception influence the acceptance of AI-based digital mourning technologies. A total of 129 valid survey responses were collected and analyzed using Partial Least Squares Structural Equation Modeling (PLS-SEM).

**Results:**

The findings indicate that performance expectancy, effort expectancy, social influence, and ethical concern significantly predict users' intention to adopt digital mourning technologies. Additionally, grief perception not only influences adoption intention but also directly affects actual usage behavior.

**Discussion:**

This study highlights that the acceptance of AI-based digital mourning technologies extends beyond instrumental rationality. It is shaped by the interplay of emotional vulnerability and moral tension. The results contribute to a deeper understanding of the ethical and psychological dimensions of posthumous AI applications and provide valuable insights for future human-AI interaction design, digital commemoration systems, and the governance of end-of-life technologies.

## 1 Introduction

Digital mourning, as an emerging application of AI technology in end-of-life care, has gained traction as a form of commemorative practice following the death of cancer patients. This phenomenon encompasses a variety of technological forms—including AI-based digital replication (1), virtual reality (VR) memorial spaces, immersive interaction (2), and chatbots (3)—allowing bereaved family members to engage with the “digital identities” of deceased individuals within virtual environments. These technologies not only redefine traditional experiences of death but also reconstruct the cultural and psychosocial landscape of mourning itself (4).

**Figure 1:**
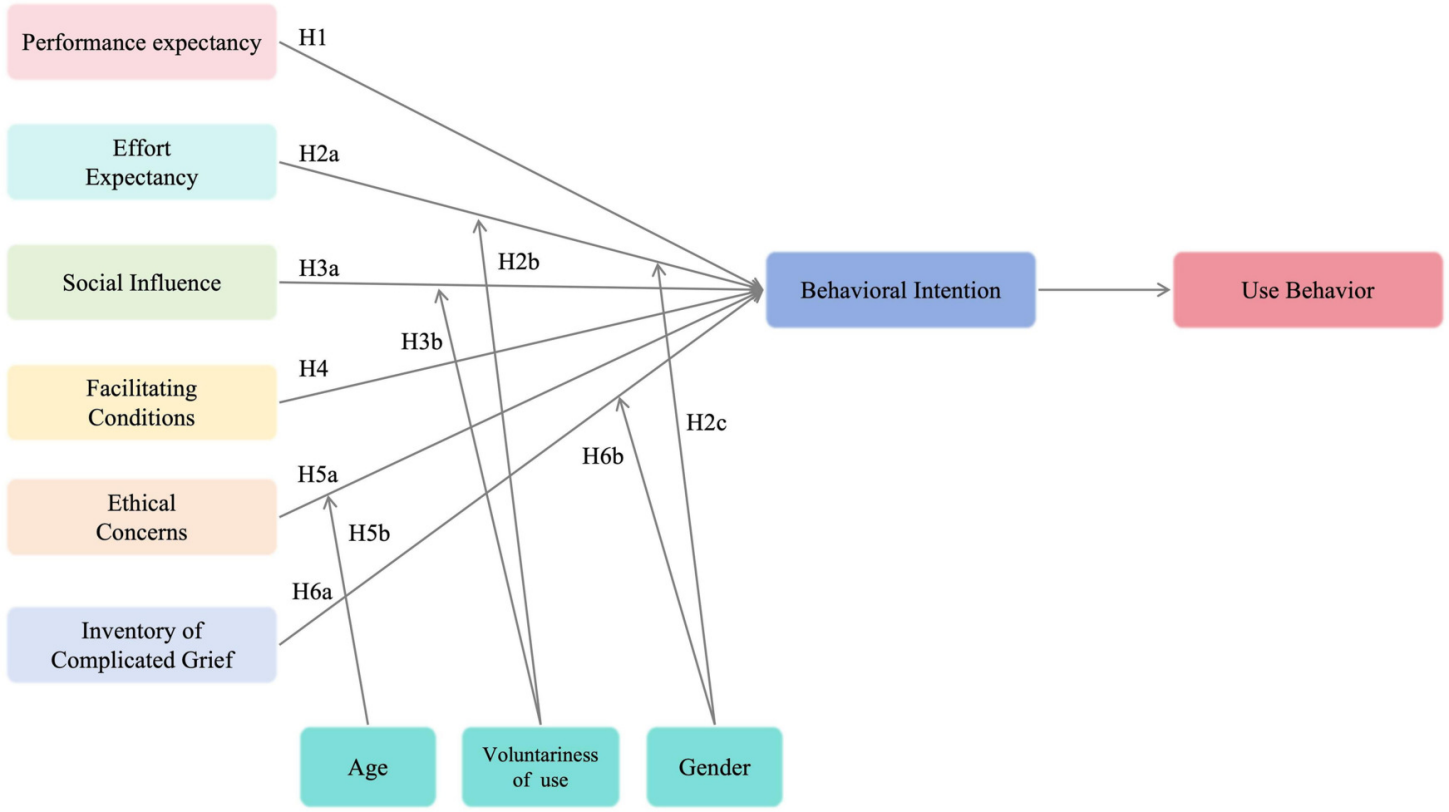
A proposed UTAUT model of AI acceptance among family members of deceased cancer patients.

**Table 1:** Descriptive statistics of respondent demographics.

Variable	Options	*n*	%
Gender	Male	61	47.29%
	Female	68	52.71%
Age	Lower 18	5	3.88%
	18–25	80	62.02%
	26–30	26	20.16%
	31–40	11	8.53%
	41–50	4	3.10%
	51–60	3	2.33%
	More than 60	0	0.00%
Education	Junior high school or below	1	0.78%
	Senior high school/Vocational school	9	6.98%
	University/Bachelor's degree	57	44.18%
	Postgraduate degree or above	62	48.06%

**Table 2:** Convergent validity indicators for latent constructs (factor loadings, AVE, CR, cronbach's alpha).

Variables	Specific question items	Factor loading	Cronbach's alpha (CA)	Composite reliability (CR)	AVE
Behavioral intention (BI)	BI1	0.907	0.833	0.901	0.752
	BI2	0.877			
	BI3	0.815			
Ethical concern (EC)	EC1	0.905	0.884	0.928	0.811
	EC2	0.906			
	EC3	0.892			
Effort expectancy (EE)	EE1	0.875	0.839	0.919	0.850
	EE2	0.967			
Facilitating conditions (FC)	FC2	0.930	0.948	0.966	0.906
	FC3	0.963			
	FC4	0.962			
Performance expectancy (PE)	PE1	0.939	0.944	0.964	0.899
	PE2	0.959			
	PE3	0.946			
Social influence (SI)	SI1	0.911	0.781	0.901	0.820
	SI4	0.900			

**Table 3:** Discriminant validity assessment based on Fornell–Larcker criterion.

Construct	AGE	BI	ICG	EC	EE	FC	GDR	PE	SI	UB	Vuse
AGE	1										
BI	0.099	0.867									
ICG	0.05	0.433	1								
EC	−0.041	0.701	0.363	0.901							
EE	−0.078	−0.167	−0.41	−0.177	0.922						
FC	−0.08	0.399	0.413	0.387	−0.117	0.952					
GDR	−0.132	−0.004	0.103	0.051	−0.049	0.106	1				
PE	0.154	0.655	0.351	0.562	−0.26	0.2	−0.047	0.948			
SI	−0.019	0.593	0.271	0.548	−0.199	0.229	−0.023	0.464	0.906		
UB	0.067	0.758	0.447	0.607	−0.239	0.208	0.015	0.673	0.466	1	
Vuse	0.02	0.775	0.375	0.69	−0.188	0.339	−0.09	0.658	0.532	0.774	1

**Table 4:** Collinearity statistics of the structural model (VIF).

Specific items	VIF
EC1	2.376
EC2	2.613
EC3	2.548
EE22	2.093
EE33	2.093
FC1	2.826
FC2	3.505
FC3	3.947
GDR	1.000
PE1	3.237
PE3	3.473
PE4	2.284
SI1	1.695
SI4	1.695
UB	1.000
Vuse	1.000

While these AI products can simulate the deceased's behaviors and responses based on personal data, constructing a “ghost”-like digital mourning form through inference and prediction—thereby introducing novel support for emotional continuity and adaptive grief coping (5), they simultaneously generate a series of ethical tensions and psychosocial risks. Notably, algorithmic simulations of the deceased blur the ontological boundaries between life and death (6), potentially causing cognitive disconnection from mortality among the bereaved.Furthermore, AI-mediated mourning may foster a commercialized “affective outsourcing” (7)—where mourners’ subjectivity becomes increasingly co-constituted, even subordinated to mechanical processes of memory management and emotional regulation. These developments compel a reexamination of two fundamental questions: What constitutes authentic grief? And to what extent can mourning—once a private, human-centered process—be technologized without compromising its existential significance and moral core?

In terms of form, digital mourning technologies provide more diverse avenues for memorialization, particularly under the integration of AI and virtual reality, where their roles in emotional companionship and memory reconstruction have gained increasing attention. However, for family members of cancer patients—who often endure prolonged caregiving and emotional exhaustion—this process may not signify healing; rather, it may exacerbate both ethical dilemmas and grief perception.

Cancer typically entails a slow and irreversible process of bodily deterioration, often accompanied by intense pain, a sense of medical futility, and the erosion of personal dignity (8). Family members, in such contexts, frequently undertake multiple roles: as emotional companions, caregiving executors, and ethical proxies in medical decision-making (9). The emotional burdens accumulated during this period rarely dissipate after the patient's death; instead, they often manifest in highly complex grief experiences—such as prolonged sadness, guilt, moral distress, or even post-traumatic symptoms.

Against this backdrop, the introduction of digital mourning technologies—such as AI-based replication and VR memorials—though envisioned as tools for emotional connection and memory continuity, may present unique ethical and psychological challenges for cancer-bereaved families. On one hand, digital identities are typically constructed from limited pre-death data and are prone to distortion or recomposition during algorithmic generation (10). The inconsistencies between replicated personas and real memories may create identity dissonance and a rupture in the sense of authenticity (11). On the other hand, for those whose emotional wounds from caregiving remain unhealed, the AI-mediated reproduction of the deceased's voice, image, or interactive behavior—while seemingly offering comfort (12)—may inadvertently trigger emotional flooding, grief recurrence, or even psychological retraumatization (13).

Moreover, cancer care often involves highly moralized decisions such as “when to let go” or “whether to prolong life,” making the technical reconstitution of the deceased a potential catalyst for renewed existential reflection—Has death truly occurred? Has mourning reached completion? These questions evoke deeply entangled experiences of ethical unease (14) and grief perception (15).

Therefore, for bereaved family members of cancer patients, digital mourning is not merely a matter of behavioral adoption of new technologies. Rather, it constitutes a psychosocial mechanism at the intersection of ethical judgment, emotional processing, and technological identity. This constitutes the theoretical starting point of the present study.

While existing literature has primarily focused on the emotional and technical feasibility of such technologies, there remains a critical lack of analysis on how bereaved families conceptualize the interrelation between technology, ethics, and grief. In particular, the mechanisms through which grief experience interacts with ethical tensions in digital mourning have yet to be systematically theorized. The relationship between digital technologies and moral norms is complex and mutually constitutive. Technologies not only shape values and environments but are themselves embedded in and shaped by normative frameworks—a core focus of ethical analysis (16, 17).

To address these gaps, the present study constructs a technology acceptance model for bereaved family members based on the Unified Theory of Acceptance and Use of Technology (UTAUT). It incorporates Foucault's theory of subjectivation and phenomenological-ethical inquiry to critically frame the psychological and normative dimensions of digital mourning. By introducing ethical conflict perception and grief perception (ICG) as independent variables, this study seeks to empirically examine the extent to which AI-mediated mourning is accepted by bereaved family members of cancer patients.

## 2 Literature review and research hypotheses

### 2.1 Unified theory of acceptance and use of technology (UTAUT)

The Unified Theory of Acceptance and Use of Technology (UTAUT) was introduced by Venkatesh and colleagues in 2003. The main goal of this model was to combine the strengths of various previous models related to technology acceptance. By doing this, UTAUT aimed to improve the ability to explain and predict why users accept and use technology, as well as how they behave when using it. UTAUT integrates eight earlier models, including the Technology Acceptance Model (TAM), Theory of Planned Behavior (TPB), and Innovation Diffusion Theory (IDT), among others. It establishes a core framework based on performance expectancy, effort expectancy, social influence, and facilitating conditions, while incorporating gender, age, experience, and voluntariness as moderating variables to account for differences in technology acceptance across demographic groups (18). Subsequently, numerous scholars have extended the UTAUT framework by integrating contextual factors, such as cultural influence (19, 20), perceived risk (21), trust (22), and users’ emotional responses (23, 24).

Since its inception, UTAUT has been widely applied across a variety of domains due to its strong predictive capabilities, including education (25), healthcare (26), e-government (27), fintech (28), and mobile internet (29). To further enhance its predictive scope, Venkatesh, Thong and Xu (23) proposed UTAUT2 adding new constructs such as hedonic motivation, price value, and habit to better account for technology adoption in consumer contexts. Many scholars have since built upon the UTAUT framework by integrating aspects like cultural influences, perceived risk, trust, and users’ emotional responses. This has led to the model's enrichment across various academic fields and cultural contexts. These advancements have substantially deepened the theoretical understanding of UTAUT and broadened its practical relevance.

In recent studies, the UTAUT has been increasingly employed to explore user acceptance of emerging digital technologies such as artificial intelligence (30) and virtual reality (31). However, our review of current studies indicates that existing applications of the model often overlooks the ethical and emotional dimensions of technology acceptance. To address this gap, this study proposes an innovative extension of the UTAUT framework, demonstrating that the model not only effectively captures rational acceptance behavior but can also be integrated with variables related to emotions, ethics, and perceived risks to uncover the deeper psychological drivers behind technology adoption. Moving forward, as technological progress becomes more intertwined with social and ethical concerns, the continued integration and development of the UTAUT model will remain highly valuable both in theory and in practice.

### 2.2 Ethical issues in digital mourning

With the rapid advancement of artificial intelligence technologies, digital mourning has emerged as a novel form of commemoration and has been increasingly integrated into practices of end-of-life care and funerary culture (12). For instance, through AI-based replication, virtual memorial spaces, and voice-interactive systems, bereaved families can engage in immersive interactions with so-called “deathbots” representing the deceased (32).Specifically, we now categorize ethical issues into four interrelated dimensions, each supported by recent scholarly literature:

Identity Authenticity: AI-generated simulations may misrepresent the deceased's moral character, personality, or social roles, leading to a distortion of memory (33). Consent Ambiguity: Most platforms lack mechanisms for pre-mortem consent regarding digital data usage, creating unresolved issues around authorization (34). Emotional Manipulation: Extended AI-mediated interactions may cultivate emotional dependency, intensifying grief instead of alleviating it (35). Posthumous Data Rights: The commodification of digital remains has triggered ownership disputes between bereaved families and commercial providers (34).

Drawing on Foucault's concepts of disciplinary power and subjectivation (36), these technologies—while ostensibly therapeutic (37)—can standardize and regulate grieving behaviors. This creates a form of “programmed grief,” where personal mourning becomes shaped by algorithmic design. As a result, the mourner's agency is displaced by technologically scripted responses, diminishing autonomy and reducing mourning to a reactive process. In this context, digital mourning functions not simply as a commemorative tool, but as a subtle apparatus of governance within the digital surveillance environment (38).

While digital mourning offers new mediums for emotional expression and psychological comfort, it also raises a host of ethical concerns—particularly in the domains of data privacy, AI-based personhood simulation, and emotional manipulation (1). Furthermore, the right to individualized mourning (39) remains ill-defined, and empirical studies on these topics are still sparse (40). Consequently, measuring users’ ethical awareness—particularly whether they perceive digital mourning as a potential overreach into sensitive posthumous data—can reflect the tension between technological trust and moral anxiety.

Beyond data privacy, a more contentious issue lies in the ethical legitimacy of reconstructing a deceased person's identity via AI (41). Some platforms train large language models capable of mimicking the deceased's speech patterns, behavioral preferences, and even generating personalized responses (42), leading to what may be described as “simulated personhood.” While these AI systems are often branded with narratives of “continued existence,” a fundamental ethical question persists: are these systems genuine extensions of the deceased, or merely algorithmic performers? This ambiguity poses risks of eroding posthumous dignity, potentially undermining the very notion of “honoring the dead” (41). Moreover, the illusion of real continuity may interfere with healthy grief processing: users may become emotionally attached to AI-generated surrogates, leading to delayed psychological detachment, emotional dependency, or identity confusion (32). Thus, while such systems simulate connection, they may disrupt the natural course of mourning and reshape individuals’ perceptions of death itself (12).

Despite their therapeutic claims, digital mourning platforms may engage in subtle forms of emotional governance. Their design often includes automated prompts—like birthday reminders or holiday messages—embedded with therapeutic intent (43). Yet these algorithmic interventions shape users’ grief trajectories, potentially overriding personal timelines (44)). This raises a critical question: are these features truly tailored to individual grieving needs, or do they reflect a broader tendency toward the technological standardization of mourning? If perceived as excessive or manipulative, these interactions may erode user trust and reduce the likelihood of technology adoption. Consequently, perceived ethical tension may emerge as a key determinant of behavioral intention—warranting its integration into extended UTAUT models.

### 2.3 Grief perception and bereavement experience

In the context of illness-related death—especially in cases of cancer, where the disease is protracted, the process of decline is gradual yet evident, and the caregiving burden is particularly heavy—the psychological responses associated with bereavement tend to be significantly more complex than those triggered by sudden death. Prior studies indicate that family members bereaved by cancer often experience elevated levels of psychological distress, including symptoms of depression and anxiety, which are closely linked to their perceived suffering of the patient during the end-of-life period (45, 46). These family members commonly experience a prolonged emotional process that includes diagnosis, treatment, decline, and ultimately, death. This journey is characterized by anticipatory grief (47), anxiety related to ethical decision-making (48), and self-sacrificing caregiving actions (49), all of which frequently develop into profound grief after the loss (50). This grief is not a simple feeling but rather a complex psychological condition involving sadness, denial, anxiety, loneliness, and guilt. Its strength and how long it lasts can go well beyond typical grieving patterns and may appear as complicated grief (51).

Complicated grief, also known as prolonged grief disorder or delayed mourning, has been strongly associated with major depressive disorder, post-traumatic stress disorder (PTSD), and significant difficulties in social interactions (52). In some instances, it can worsen PTSD symptoms (53). This condition is frequently marked by an inability to let go of the deceased, denial of the death, ongoing difficulties in managing emotions, and the breakdown of life goals and trust in others (54–56). As Lichtenthal and colleagues have pointed out, for those who cared for cancer patients, grief is not just an emotional response. It often stems from the loss of their identity as a caregiver, their sense of ethical control, and how they see themselves in relation to others—leading to a type of grief that disrupts their sense of self, is hard to express, and deeply unsettling (57).

In recent years, researchers have increasingly focused on how people's understanding and experience of grief affect their behavior (58–61). Instead of only seeing grief as a result, a growing amount of research now considers how individuals perceive grief—often measured using the Inventory of Complicated Grief (ICG)—as a cognitive and emotional factor that can influence whether they adopt new technologies, participate in social activities, and make decisions involving risk (62). Specifically, when it comes to technologies used at the end of life and AI tools for remembrance, a person's individual experience of suffering can significantly shape how they think and evaluate things, their moral judgments, and the choices they make. For instance, some studies have found that whether people are willing to accept AI technologies for mourning is closely linked to their emotional ability to cope and how they interpret grief. Those who feel emotionally resistant or have ethical doubts tend to be less willing to use these technologies (63, 64).

In the case of immersive digital mourning technologies, this psychological mechanism becomes especially complex. On one hand, these technologies can provide spaces for ongoing emotional connection and the preservation of memories, and are often viewed as ways to ease grief and strengthen the feeling of closeness with someone who has passed away (65). On the other hand, they might reawaken unresolved emotional pain, potentially trapping individuals in a repetitive cycle of technological mourning (66). In their assessment of VR-based grief interventions, Pizzoli et al. (2) discovered that individuals with high scores on the Inventory of Complicated Grief (ICG) were more likely to experience cognitive dissonance and a blurring of reality when interacting with AI-generated representations of the deceased. This “knowing it's artificial, but emotionally unable to let go” experience weakens the therapeutic efficacy of the technology (2). When such mechanisms intersect with AI-facilitated reanimations of the deceased, individuals may find themselves torn between the longing to reconnect and the emotional overload that compels rejection of the digital representation. These findings reinforce the view that grief perception is not a neutral background condition but a decisive antecedent variable in technology acceptance.

Accordingly, this study incorporates Perception of Complicated Grief as a key independent variable within the extended UTAUT model to predict bereaved cancer family members’ willingness to adopt AI-based digital mourning technologies. Here, we use the Inventory of Complicated Grief (ICG) (67) scale to measure perception of complicated grief. This model refinement aligns with cognitive-emotional decision-making theory, which assigns functional roles to affective variables, and responds to the unique moral-emotional entanglements of the digital mourning context. By introducing this construct, the study aims to go beyond rationalist acceptance models to offer a more psychologically grounded understanding of how grief and death experiences shape technology adoption in ethically charged domains.

### 2.4 Research questions and hypotheses

Based on the preceding literature and theoretical integration, this study aims to address the following four core research questions:

RQ1: Can the four core predictors in the original UTAUT model—performance expectancy (PE), effort expectancy (EE), social influence (SI), and facilitating conditions (FC)—effectively predict the behavioral intention (BI) and use behavior (UB) of bereaved family members of cancer patients toward AI-based digital mourning technologies?

RQ2: Building on the UTAUT model, do context-specific variables such as ethical concern (EC) and grief perception (ICG) significantly enhance the model's explanatory power? In other words, do these extended constructs contribute a statistically and theoretically meaningful increment to the prediction of behavioral intention?

RQ3: Do demographic variables (e.g., age, gender) serve as moderators between key technology perception variables and behavioral intention? How do such moderating effects reveal differentiated behavioral pathways among users facing emotionally intensive technologies?

RQ4: Is behavioral intention (BI) still the strongest predictor of actual use behavior (UB) in the context of AI commemorative systems? In other words, once users form an intention to use the technology, does it consistently translate into actual engagement?

To conduct empirical tests on these issues, the following research hypotheses are proposed. The corresponding diagrams are shown in Figure 1:

H1. Performance expectancy (PE) has a significant positive effect on behavioral intention (BI).

H2a: Effort expectancy (EE) has a significant positive effect on behavioral intention (BI).

H2b: Gender (GDR) negatively moderates the relationship between effort expectancy (EE) and behavioral intention (BI).

H2c: Voluntariness of use (Vuse) positively moderates the relationship between effort expectancy (EE) and behavioral intention (BI).

H3a: Social influence (SI) has a significant positive effect on behavioral intention (BI).

H3b: Voluntariness of use (Vuse) negatively moderates the relationship between social influence (SI) and behavioral intention (BI).

H4. Facilitating Conditions: Facilitating conditions (FC) have a significant positive effect on behavior intention (BI).

H5a: Ethical concern (EC) has a significant negative effect on behavioral intention (BI).

H5b: Age negatively moderates the relationship between ethical concern (EC) and behavioral intention (BI).

H6a: Grief perception (ICG) has a significant positive effect on behavioral intention (BI).

H6b: Grief perception (ICG) has a significant positive effect on use behavior (UB).

H6c: Gender (GDR) negatively moderates the relationship between grief perception (ICG) and behavioral intention (BI).

H7: Behavioral intention (BI) has a significant positive effect on use behavior (UB).

## 3 Research method

### 3.1 Survey method

In the early stage of questionnaire design, the research team organized a small expert consultation meeting and invited two front-line practice experts from Chongqing Medical University to participate and provide guidance. Based on clinical experience, experts have put forward targeted suggestions on issues such as the emotional responses of family members of cancer patients during the mourning process, their acceptance of technology, and possible ethical problems, and have improved the specific expression of the questionnaire. Make it more acceptable for family members. Based on the four core variables, this study added ethical care perception and pain perception as supplementary variables. The average well completion time is approximately 20 minutes. The data collection lasted for one week and a total of 137 responses were obtained. Among them, 129 were considered valid after data screening (*n* = 129).

### 3.2 Variable measurement

This study integrates the four core constructs of the Unified Theory of Acceptance and Use of Technology (UTAUT)—performance expectancy (PE), effort expectancy (EE), social influence (SI), and facilitating conditions (FC)—along with two original moderating variables (age and gender) and two additional context-specific variables: ethical concern (EC) and grief perception, measured via the Inventory of Complicated Grief (ICG). These constructs were adapted to reflect the psychological characteristics of bereaved family members of terminally ill patients. In total, six latent variables were measured.

Measurement items were developed by referencing and modifying the subdimensions of the original UTAUT scale proposed by Venkatesh et al., tailored to the specific context of bereavement and digital mourning. All constructs were measured using a five-point Likert scale, with 2–4 items per construct.

Participants (bereaved family members) were required to respond to all mandatory items. Example items included:“I believe AI-based mourning technologies can help me better commemorate my deceased loved one” (Performance Expectancy),“I find using AI mourning technologies difficult” (Effort Expectancy),“I think professionals (such as doctors or counselors) would recommend the use of AI mourning technologies” (Social Influence),“I can easily access guidance and assistance on how to use AI mourning technologies” (Facilitating Conditions).

### 3.3 Data analysis

To systematically explore the acceptance mechanisms of AI-based digital mourning among bereaved family members of cancer patients, this study employed a Partial Least Squares Structural Equation Modeling (PLS-SEM) approach. Data were analyzed using SmartPLS 27, which is well-suited for modeling complex path structures involving small samples, non-normal data, and moderated relationships.

Given the sensitivity of the study population—bereaved individuals with typically low public engagement, potential trauma triggers related to AI commemoration, ethical concerns, and limited technological exposure (1, 32)—the use of PLS-SEM is especially appropriate. The final dataset included 129 valid responses, and the Shapiro–Wilk test confirmed non-normal distribution for 43 out of 43 measurement items (*p* < 0.05). These conditions (*N* < 200 and significant non-normality) strongly justify the methodological fit of PLS-SEM, which remains robust under such constraints and does not rely on the assumption of multivariate normality.

This study first evaluated convergent validity by examining the factor loadings and average variance extracted (AVE) for each latent construct, and assessed internal consistency using composite reliability (CR). Subsequently, discriminant validity was tested using the Fornell–Larcker criterion to ensure adequate separation among the latent variables.

A structural model path diagram was generated, and the bootstrapping method was employed to assess key structural characteristics, including collinearity diagnostics, explanatory power (R^2^), model fit (SRMR), and predictive relevance (Q^2^). Finally, the significance of each hypothesized path in the extended UTAUT model was evaluated, which enabled the identification of significant relationships among the latent constructs and provided insights into the overall structural mechanism.

## 4 Digital research

### 4.1 Descriptive statistics of the sample

Following ethical approval from the Human Research Ethics Committee for Non-Clinical Faculties of Chengdu Neusoft University [Approval No. (CNU20241120)] and compliance with China's Personal Information Protection Law and institutional data governance standards, we administered the survey questionnaire distribution. A total of 207 questionnaires were distributed and collected in southern China. After manual data cleaning to remove invalid responses, 129 valid samples were retained, resulting in a valid response rate of 62.32%. Descriptive statistics of the sample were generated using SmartPLS.

Among the 129 valid respondents, 68 were female (52.71%) and 61 were male (47.29%). In terms of age distribution, the 18–25 age group constituted the majority of participants (62.02%), followed by the 26–30 age group (20.16%). Respondents aged over 50 years accounted for only 2.33% of the total sample. Demographic characteristics of the sample are summarized in Table 1.

### 4.2 Measurement model: reliability and validity assessment

This study employed the Partial Least Squares Algorithm function in SmartPLS 3.27 to evaluate the reliability and validity of each latent construct. Specifically, the analysis examined Cronbach's Alpha (CA), Composite Reliability (CR), and Factor Loadings for all items.

Validity assessment was conducted from two perspectives: convergent validity and discriminant validity. For convergent validity, the Average Variance Extracted (AVE) was calculated for each construct to assess the extent to which items reflect the intended latent variable. Discriminant validity was evaluated by comparing the square root of each construct's AVE with its correlations with other constructs, in accordance with the Fornell–Larcker criterion (68). Convergent validity results are detailed in Table 2, and Discriminant Validity results are presented in the Table 3.

The measurement model demonstrated satisfactory reliability, convergent validity, and discriminant validity through rigorous statistical validation. All constructs exhibited strong internal consistency (Cronbach's *α* > 0.70) and convergent validity (AVE >0.50), aligning with thresholds defined by Hair et al. (68). Discriminant validity was confirmed through established criteria (e.g., HTMT ratios <0.85), ensuring distinctness among latent variables.

Moreover, the square roots of the AVE values for each construct were greater than their correlations with other constructs, and all factor loadings were higher than their respective cross-loadings—thus fulfilling the Fornell–Larcker criterion for discriminant validity *(*69).

The measurement model aligns with established psychometric standards for reliability, convergent validity, and discriminant validity, ensuring rigorous methodological grounding for the structural model's evaluation.

### 4.3 Structural model evaluation

After validating the measurement model, the study proceeded to examine the structural model, focusing on the model's predictive power and the causal relationships among latent constructs. The structural model was tested using SmartPLS 3.27, employing the bootstrapping procedure. The evaluation process included the following four steps:

(1) Collinearity assessment: Variance Inflation Factor (VIF) values were calculated to evaluate multicollinearity and the model's structural stability.
(2) Explanatory power: The Coefficient of Determination (R^2^) was used to assess how well the exogenous constructs explained the variance in the endogenous variables.
(3) Model fit: The Standardized Root Mean Square Residual (SRMR) was calculated as an index of model fit.
(4) Predictive relevance: The Construct Cross-Validated Redundancy (Q^2^) was computed to evaluate the predictive relevance of the structural model (70).

These four indicators jointly assess the adequacy, explanatory power, and predictive performance of the model. In addition, the analysis of path coefficients, as well as direct and indirect effect sizes, was conducted to further evaluate the relationships among latent constructs. This step enables the study to address the research questions, test the proposed hypotheses, and determine the relative contribution of each independent variable to the acceptance of AI-based mourning technologies among bereaved family members.

According to the PLS-SEM framework, the model includes the following variables:

Exogenous latent constructs: Performance Expectancy (PE), Effort Expectancy (EE), Social Influence (SI), and Facilitating Conditions (FC).

Endogenous latent constructs: Behavioral Intention (BI) and Use Behavior (UB).

Observed moderating variables: Age, Gender, and Voluntariness of Use (Vuse).

Together, these components form the structural model used to explain and predict acceptance behavior toward AI-driven digital mourning technologies among family members of deceased cancer patients.

#### 4.3.1 Collinearity diagnostics

In Partial Least Squares (PLS) data analysis, the Variance Inflation Factor (VIF) serves as a critical indicator for assessing potential multicollinearity within the structural model. As defined by Hair et al. in the context of SmartPLS-based modeling, a VIF value of 5 or higher indicates serious multicollinearity, whereas a VIF value of 3 or higher may suggest potential multicollinearity concerns that warrant further scrutiny (71).

As shown in the Table 4, all VIF values for the latent constructs in the model are below the threshold of 5, indicating that there is no severe multicollinearity among the variables. This finding validates the rationality of the questionnaire design, particularly the construct-specific item development strategy. Moreover, it suggests that the questionnaire items effectively differentiate between distinct latent dimensions, thereby minimizing the risk of estimation bias or model distortion caused by collinearity.

#### 4.3.2 Evaluation of explanatory power

PLS-SEM employs ordinary least squares (OLS) regression to estimate path coefficients and factor loadings, aiming to maximize the explained variance (R^2^) of endogenous constructs. This approach is particularly suitable for complex models and small samples, effectively capturing causal relationships among latent variables. According to Hair et al., the explanatory power of structural models can be categorized into three levels: R^2^ ≥ 0.75 (substantial), 0.50 (moderate), and 0.25 (weak) (72).

As shown in the Table 5, the R^2^ value for Behavioral Intention (BI) is 0.770, indicating that exogenous variables such as performance expectancy and effort expectancy collectively explain 77.0% of the variance in BI. This exceeds the typical explanatory power observed in conventional UTAUT applications, which usually ranges between 50% and 60%. The adjusted R^2^ value of 0.745 further confirms the model's explanatory strength even after accounting for degrees of freedom, suggesting that the model is robust with respect to both variable count and sample size.

**Table 5:** Coefficient of determination (R^2^).

Endogenous variable	R-square	R-square adjusted
BI	0.770	0.745
UB	0.614	0.605

**Table 6:** Standardized root mean square residual (SRMR).

Model type	Saturated model	Estimated model
SRMR	0.077	0.078

Similarly, the R^2^ value for Use Behavior (UB) is 0.614, with an adjusted R^2^ of 0.605. This indicates that the model explains 60.5% of the variance in actual use behavior, reflecting a relatively high level of explanatory power even after considering the interrelationships among the variables.

Taken together, these results demonstrate that the model possesses strong predictive capacity for the endogenous variables, supporting its validity for explaining user acceptance of AI-based applications in emotionally complex domains such as digital mourning.

#### 4.3.3 Model fit evaluation

This study adopted the Standardized Root Mean Square Residual (SRMR) to assess the overall model fit. According to the criteria proposed by Henseler and Sarstedt, an SRMR value below 0.14 indicates acceptable model fit. The SRMR value of 0.078 (Table 6) indicates good model fit (73).

#### 4.3.4 Predictive relevance (Q^2^) evaluation

Predictive relevance (Q^2^) is a key indicator in PLS-SEM used to assess the model's predictive validity. The Q^2^ value ranges from negative infinity to 1, with higher values indicating stronger predictive relevance. In this study, the PLSpredict procedure was applied to compute Q^2^ values. As shown in Table 7, the Q^2^ values for the two endogenous latent variables were Behavioral Intention (BI) = 0.673 and Use Behavior (UB) = 0.613. Since both values are greater than zero, the results confirm that the exogenous constructs in the model exhibit adequate predictive relevance for the endogenous constructs.

**Table 7:** Predictive relevance (Q^2^) results for the structural model.

Endogenous variable	Q^2^predict
BI	0.673
UB	0.613

## 5 Hypothesis testing results

This study applied bootstrapping with 5,000 resamples to estimate the path coefficients and assess their statistical significance within the structural model. The significance threshold was determined by T-statistics greater than 1.96 and *p*-values less than 0.10, with *p* < 0.05 being considered the standard for robust significance. The validity of each hypothesis was evaluated based on these criteria.

**Figure 2:**
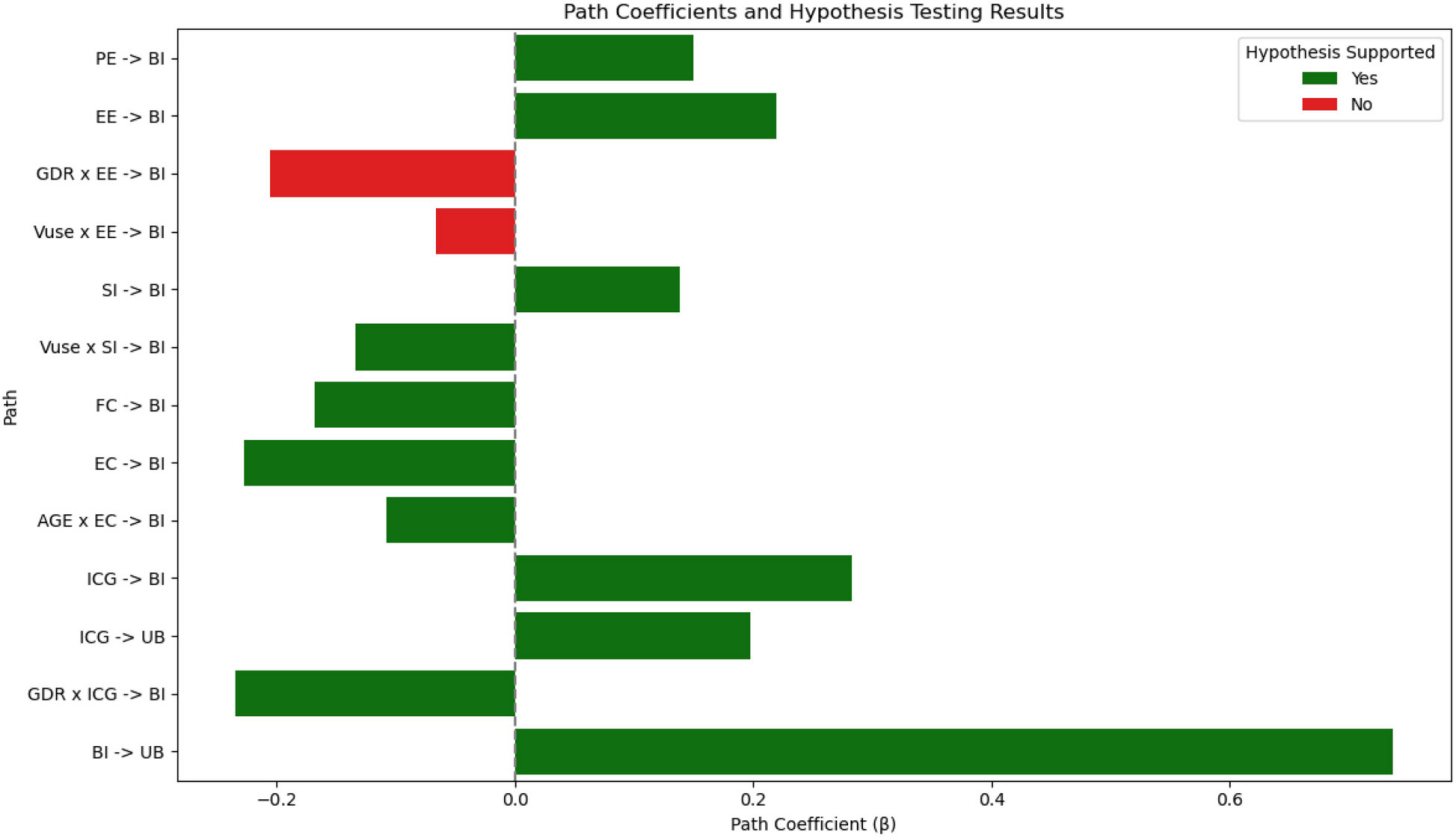
Path coefficients and hypothesis lesting results.

**Figure 3:**
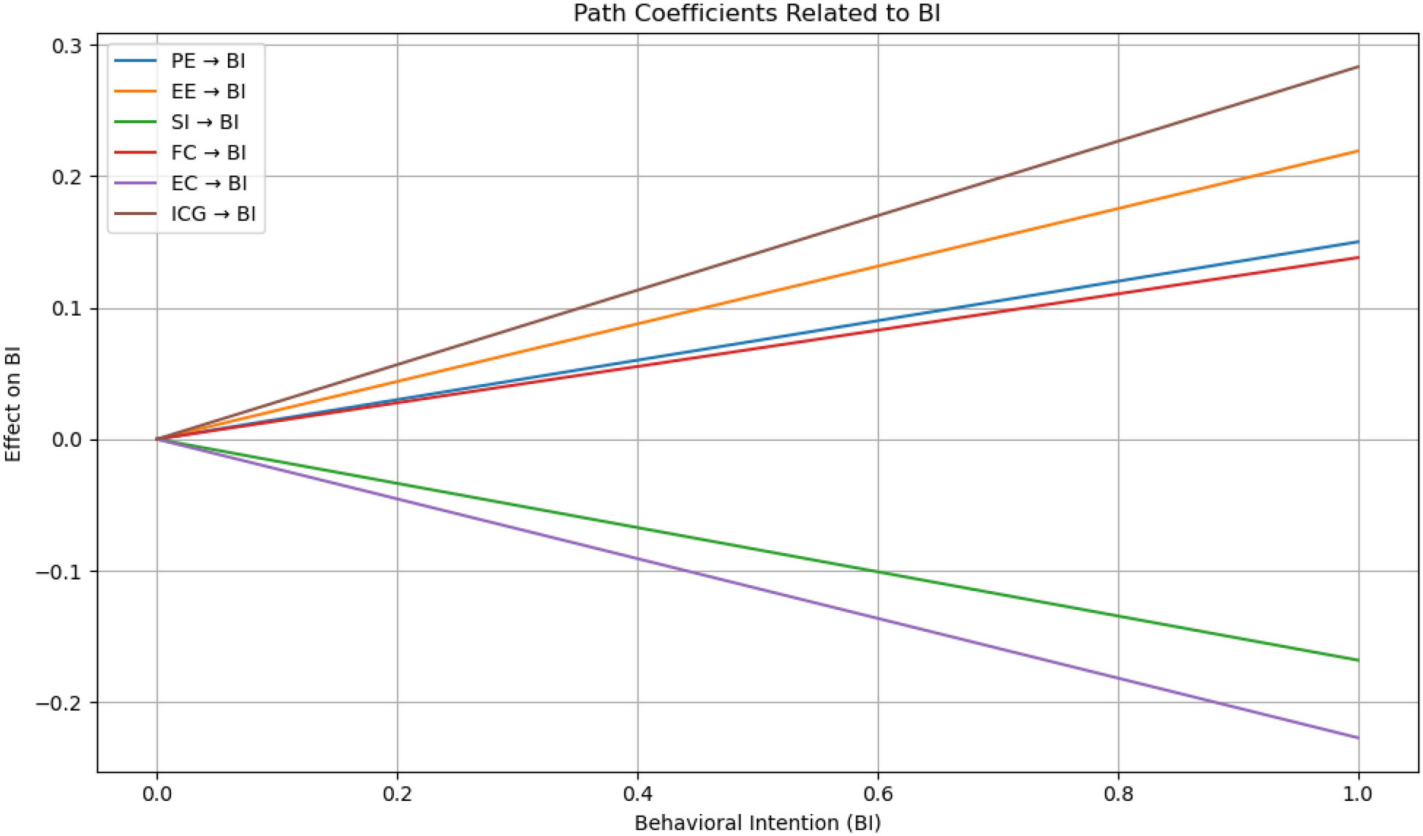
Dynamic path coefficients of behavioral intention (BI) determinants.

Additionally, the magnitude of each path coefficient indicates the relative strength of influence exerted by the independent variables on the dependent constructs. The results of hypothesis testing are summarized in Table 8, and the bar chart (Figure 2) summarizes the β coefficients and hypothesis testing results of all paths. Color coding is used to distinguish supported and unsupported hypotheses, as well as a simple slope interaction graph depicting the trajectories of behavioral intent (BI) under different independent variables (PE, EE, SI, FC, EC, ICG). It provides an intuitive understanding of path strength and directionality (see Figure 3).

**Table 8:** An extended UTAUT model of acceptance and Use of AI-based mourning technologies Among bereaved families of cancer patients.

Hypothesis	Paths	Path coefficient (β)	Sample mean (M)	Standard deviation (STDEV)	T statistics (|O/STDEV|)	*P* values	Hypothesis testing
H1	PE -> BI	0.150	0.141	0.075	2.015	0.044	Supported
H2a	EE -> BI	0.219	0.201	0.088	2.494	0.013	Supported
H2b	GDR x EE -> BI	−0.206	−0.200	0.114	1.802	0.072	Not supported
H2c	Vuse x EE -> BI	−0.066	−0.065	0.047	1.419	0.156	Not supported
H3a	SI -> BI	0.138	0.134	0.07	1.981	0.048	Supported
H3b	Vuse x SI -> BI	−0.134	−0.133	0.05	2.66	0.008	Supported
H4	FC -> BI	−0.168	−0.164	0.075	2.241	0.025	Supported
H5a	EC -> BI	−0.227	−0.226	0.08	3.386	0.001	Supported
H5b	AGE x EC -> BI	−0.108	−0.107	0.054	1.98	0.048	Supported
H6a	ICG -> BI	0.283	0.278	0.088	3.222	0.001	Supported
H6b	ICG -> UB	0.198	0.198	0.069	2.893	0.004	Supported
H6c	GDR x ICG -> BI	−0.235	−0.233	0.108	2.178	0.029	Supported
H7	BI -> UB	0.737	0.736	0.061	12.098	0.000	Supported

### 5.1 Hypotheses and interpretations

Based on the extended UTAUT model integrating both ethical and emotional variables, this study proposed a total of 13 hypothesis paths, of which 11 were statistically supported. These findings confirm that both affective and ethical factors play a critical role in shaping the behavioral intentions of bereaved family members toward AI-based digital mourning technologies.

First, performance expectancy (PE) was found to have a significant positive effect on behavioral intention (BI) (H1, *β* = 0.150, t = 2.015, *p* = 0.044), consistent with Venkatesh et al. (18), who argue that users' beliefs about the utility of a technology directly influence their intention to adopt it. In the context of digital mourning, family members who believe that AI technologies can alleviate grief or help restore emotional bonds are more inclined to accept their use.

Effort expectancy (EE) also exhibited a significant positive effect on BI (H2a, *β* = 0.219, t = 2.494, *p* = 0.013), suggesting that under emotionally intense circumstances, such as bereavement, individuals tend to value the ease of use and low emotional burden of new technologies. This is aligned with prior findings that emphasize the emotional benefits of user-friendly systems.

Social influence (SI) showed a significant impact on BI (H3a, *β* = 0.138, t = 1.981, *p* = 0.048), indicating that decisions around AI-based mourning are influenced not only by personal beliefs but also by the opinions of family, friends, and healthcare professionals. Moreover, voluntariness of use (Vuse) significantly and negatively moderated the relationship between SI and BI (H3b, *β* = –0.134, t = 2.660, *p* = 0.008), revealing that first-time users rely more heavily on external opinions, whereas more experienced users tend to form more autonomous judgments—reflecting an increase in user independence with experience.

Interestingly, facilitating conditions (FC) were found to have a significant negative effect on actual behavior Intention (BI) (H4, *β* = –0.168, t = 2.241, *p* = 0.025). While this contradicts the traditional UTAUT model assumption that facilitating conditions promote behavioral adoption, it reveals a unique dynamic within the digital mourning context.

Ethical concern (EC) had a significant negative effect on behavioral intention (H5a, *β* = –0.227, t = 3.386, *p* = 0.001), echoing discussions in Chapter 2 that ethical considerations are central to digital mourning acceptance. Additionally, age was found to negatively moderate this relationship (H5b, *β* = –0.108, t = 1.980, *p* = 0.048), suggesting that older individuals may be more sensitive to ethical issues, thereby weakening the effect of ethical concern on their intention to adopt the technology.

Grief perception, as measured by the Inventory of Complicated Grief (ICG), significantly and positively influenced both behavioral intention (H6a, *β* = 0.283, t = 3.222, *p* = 0.001) and use behavior (H6b, *β* = 0.198, t = 2.893, *p* = 0.004). This supports the emotional activation hypothesis presented in Chapter 2—namely, that individuals experiencing higher levels of grief are more likely to engage with digital tools as a form of emotional compensation.

Furthermore, gender (GDR) negatively moderated the relationship between grief perception and behavioral intention (H6c, *β* = –0.235, t = 2.178, *p* = 0.029), indicating that gender-based psychological or emotional mechanisms may reduce the impact of grief perception on decision-making. Finally, behavioral intention strongly predicted actual use behavior (H7, *β* = 0.737, t = 12.098, *p* < 0.001), confirming the robust predictive power of intention in the context of AI-assisted mourning and supporting the structural validity of the UTAUT framework.

### 5.2 Unsupported hypotheses and interpretations

Despite most paths being statistically significant, two moderating hypotheses were not supported. Specifically, the moderating effects of gender and voluntariness of use on the relationship between effort expectancy and behavioral intention did not reach significance.

The first unsupported hypothesis was H2b, which posited a negative moderating effect of gender (GDR) on the relationship between effort expectancy (EE) and behavioral intention (BI) (*β* = −0.206, t = 1.802, *p* = 0.072). Although this value approached the significance threshold, it failed to meet the statistical cutoff. This suggests that in the context of digital mourning technologies for bereaved cancer families, perceptions of technological ease-of-use did not differ significantly across genders.

The second unsupported path, H2c, tested the moderating effect of voluntariness of use (Vuse) on the EE–BI relationship and was also not significant (*β* = −0.066, t = 1.419, *p* = 0.156). This implies that participants’prior experiences with similar technologies had no substantial influence on the relationship between their perceived ease of use and intention to adopt AI mourning tools.

These two unsupported hypotheses collectively reveal that effort expectancy, as a construct of instrumental reasoning, may be less susceptible to modulation by demographic or affective variables in emotionally intense contexts.

## 6 Discussion

### 6.1 Discussion on path assumptions

In this study, Hypothesis H1 is supported: Performance Expectancy (PE) exerts a significant positive effect on Behavioral Intention (BI), aligning with the original UTAUT model and indicating that users are more inclined to adopt AI-based digital mourning technologies when they believe such tools can effectively alleviate grief. This finding is consistent with Davis's (74) foundational insight that PE serves as a core driver of technology acceptance, often showing strong β correlations ranging from 0.63 to 0.85. However, the β value observed in this study falls below the typical range reported in UTAUT2, where Venkatesh et al. (23) noted that PE→BI path coefficients commonly exceed 0.3. This suggests that in the context of digital mourning, the perceived functional value of technology is subordinated to emotional needs, mirroring a similar attenuation trend observed in studies of medical AI (75).

For Hypothesis H2a, the positive impact of Effort Expectancy (EE) on BI reaffirms the foundational framework of UTAUT, suggesting that improvements in usability can directly enhance acceptance intention. This aligns with findings from the TAM2 extension, where EE typically influences BI indirectly via cognitive instrumental processes. However, both H2b and H2c, which test the moderating roles of gender and user experience on EE respectively, are not supported. This contradicts the original UTAUT model's conclusion that “gender moderates EE” (18). A plausible explanation lies in the emotional intensity of mourning behaviors, which may diminish individual differences, a pattern consistent with Li et al.'s (2023) findings in AI-mediated mental health contexts.

Hypothesis H3a, examining Social Influence (SI), is also supported, suggesting that normative pressure from friends, family, or society plays a facilitating role in the adoption of AI mourning technologies. Notably, H3b—which tests the interaction effect of user experience and SI on BI—is significant and negatively signed. This implies that more experienced users are less susceptible to social influence, which aligns with Venkatesh et al.'s (2003) moderation logic: experienced users tend to rely more on their autonomous judgment than on external cues.

Hypothesis H4 regarding Facilitating Conditions (FC) is supported, with a negative path coefficient indicating that environmental or resource-related obstacles (e.g., limited access to digital services) significantly reduce behavioral intention (BI). This reinforces the core UTAUT assumption that FC affects either BI directly or Use Behavior (UB) indirectly. However, the absolute β value is lower than that reported in some revised models. For instance, Dwivedi et al. (19) reported a path coefficient of approximately −0.34 for FC→BI. That indicates, usage of emotionally sensitive technologies, such as AI commemoration systems, may depend more on an individual's psychological readiness than on practical resources like access to devices or training. Even with available support, unresolved grief or ethical concerns can hinder actual use. Conversely, focusing heavily on the technical aspects of these systems might evoke negative emotional reactions or ethical objections, thereby reducing the likelihood of their adoption. These findings indicate that promoting the acceptance of these technologies requires attention to both practical support and users’ emotional states, as well as ensuring that the technology aligns with their values.

Contrary to classical UTAUT findings (76), this study observed the disappearance of gender's moderating effect on the relationship between effort expectancy (EE) and behavioral intention (BI). This deviation may stem from the intense psychological distress inherent in cancer-related bereavement (45), which potentially overrides gender-specific behavioral patterns. Under such high-emotional-intensity conditions, both male and female bereaved individuals prioritize emotional security and existential authenticity over operational convenience, leading to a homogenization of technology evaluation criteria. This aligns with Suo et al.'s (2025) proposition that grief contexts neutralize gender disparities through an emotional homogenization effect.

Furthermore, voluntariness of use (Vuse) failed to moderate the EE→BI path—a finding resonant with Harbinja's ethical legitimacy threshold theory: “Users must first cross an ethical legitimacy threshold before evaluating usability in emotionally high-risk technologies” (77). This underscores that in digital mourning—a domain characterized by affective and ethical salience—utilitarian factors (e.g., ease of use) become secondary to existential concerns. The result corroborates Attuquayefio and Addo's (78) revised UTAUT framework, wherein moderating effects attenuate in high-stakes contexts. Digital mourning thus operates as an affective boundary condition, diminishing demographic sensitivity to functional attributes.

### 6.2 Principal findings

This study constructs an extended technology acceptance model for digital mourning within the UTAUT framework by incorporating two new variables: Perceived Grief (ICG) and Ethical Perception (EC). The empirical findings reveal a systematic transformation of traditional moderation mechanisms under high-sensitivity contexts. The theoretical contributions can be summarized in two key areas:

a. Reconfiguration of Acceptance Hierarchies Driven by Technology Sensitivity:
  Classic UTAUT theory posits that demographic variables such as gender, age, and user experience exert significant moderating effects on the core acceptance paths (18). However, our study finds that such traditional moderators lose explanatory power in emotionally sensitive contexts. Specifically, gender does not significantly moderate the path between Effort Expectancy (EE) and Behavioral Intention, while user experience negatively moderates the path from Social Influence (SI) to Behavioral Intention. This directly contradicts findings in consumer technology contexts, where experience tends to reinforce social conformity (23). This paradox can be interpreted through the lens of Technology Sensitivity Theory: when technologies intervene in emotionally charged scenarios (e.g., mourning, healthcare), users shift from a “function-first” to an “emotion-ethics-first” decision logic. As a result, demographic moderators become selectively operative only along emotion-ethical pathways, forming a context-dependent moderation filtering mechanism (75). Correspondingly, our findings show that age significantly strengthens the inhibitory effect of ethical perception, while gender attenuates the motivational effect of grief perception—indicating a reversal of traditional functional moderators. These findings challenge the universal applicability of UTAUT's moderation logic and propose new theoretical standards for researching high-sensitivity technologies.
b. The Emotional Authenticity Paradox and Ethical Intergenerational Effects in AI Mourning Technology Acceptance:
  This study also identifies two distinctive moderation effects absent from prior research: the emotional authenticity paradox and the ethical intergenerational effect. First, the negative moderation of social influence by usage experience (H3b) indicates that individuals with more digital mourning experience exhibit greater resistance to socially normative persuasion. This finding stands in sharp contrast to educational technology research, where increased experience tends to enhance social compliance (79). This divergence may stem from the inherently private nature of mourning: as users accumulate technological experience, they develop an awareness of emotional autonomy, becoming increasingly vigilant toward external interventions that might compromise the authenticity of their grief.
  Second, the study reveals a pronounced intergenerational ethical effect: age exerts a stronger negative moderation on ethical perception than on traditional predictors such as Effort Expectancy (typically |*β*| < 0.05). Older users tend to prioritize ethical boundaries over functional convenience in technology adoption decisions. This aligns with findings by Li et al. (80), who observed that “digital natives” focus more on usability, whereas “digital immigrants” emphasize ethical limits. These insights suggest the need to recalibrate UTAUT's moderation mechanism by incorporating an “ethical weighting coefficient” for age-related analyses in morally sensitive technological contexts.

### 6.3 Technical governance and suggestions

In terms of Chinese law, the data of the deceased is regarded as an object of property rights (Article 994 of the Civil Code), but the essence of digital mourning is to maintain the emotional connection between the living and the deceased. Therefore, the “maintaining connection” principle proposed by Chen Xiyi can be drawn upon to establish a “special management right for digital Remains” (81). The immediate family members of the deceased can be regarded as default managers to exercise data access rights in private mourning Spaces. When it comes to public mourning, a multi-party consultation committee should be established to balance personal emotions and public interests. This mechanism can draw on the transitional arrangements of the European Union for deadbots (82), but it places more emphasis on the sustainability of the relationship rather than the disposal of the heritage.

At the social level, it is also very important to cultivate certain pre-social resilience. Incorporate the “empathy network” into the public crisis response system, such as opening digital mourning entrances after major accidents, or developing and advocating digital life education courses to guide young people to understand the boundaries of AI mourning technology first.

At the level of digital application, medical AI retains the “non-algorithmic” emotional space of doctor-patient interaction. Digital mental health tools should set protection thresholds for the mourning process to replace automated processes and avoid the formation of “cognitive dilemmas”. An adaptive interface for the mourning stage can also be developed. Users’ usage rights can be set to expand step by step based on the duration of use. First-time users cannot directly access all AI mourning services. The platform will proactively guide users to reach a moral consensus and improve the moral mechanism.

Furthermore, the research suggests that the deceased could sign an agreement during their lifetime to prohibit commercial or non-commercial digital revivals. For historical figures, certain ethical reviews are conducted through relevant experts and scholars.

## 7 Conclusion

### 7.1 Summary of key findings

This study used the UTAUT model to systematically investigate how bereaved family members accept and use AI-based digital mourning technologies. By adding ethical concerns and grief perception to the model and using PLS-SEM for data analysis, the research demonstrated that perceived usefulness, perceived ease of use, social influence, ethical considerations, and emotional distress significantly affect both the intention to use and the actual use of these technologies. The study also found that age, gender, and whether the use of the technology was voluntary or not, influence this acceptance in complex ways, highlighting the many factors that affect technology adoption in emotionally charged situations.

Going beyond these statistical results, the study uses Foucault's theories on how individuals become subjects to interpret digital mourning not just as a tool for coping with emotions, but also as a system that can shape behavior. AI commemoration technologies provide personalized ways to remember the deceased and offer emotional support, but they also subtly guide mourning into a digital practice that is structured by computational processes, interactions, and ongoing engagement. Consequently, the bereaved individual, who once expressed grief spontaneously, increasingly becomes a ‘user’ within a technological framework, with their mourning process and emotional pace influenced by the logic of these platforms. Digital mourning, therefore, serves not only as a source of comfort but also as a subtle mechanism of control.

### 7.2 Limitations and **f**uture work

In this study, the dominance of young participants (aged 18–30) inherently limited the ability of the research to capture intergenerational dynamics in mourning practices. The specific reason for this study is that the elderly often have deeper intergenerational traumatic memories, giving mourning behavior the significance of “family continuity”, and they have a poor acceptance of the research questionnaire during the investigation period. Influenced by the trend of personalization, the youth group pays more attention to self-repair. Therefore, in the process of filling out the questionnaire, the proportion of the youth group is relatively large. This imbalance introduces a potential selection bias, favoring perspectives centered on individualistic coping and self-repair, which may not fully represent the communal or legacy-oriented mourning practices often observed among older adults. Future research must prioritize developing culturally sensitive and accessible methodologies (e.g., qualitative interviews, facilitated discussions, or alternative data collection formats) specifically designed to engage elderly populations and capture the richness of their grief experiences, particularly concerning intergenerational trauma and the meaning of “family continuity.”

Future research should expand this model's cultural and contextual adaptability, incorporating interdisciplinary perspectives to explore how digital mourning may be personalized and ethically sensitive in AI-dominated environments. Questions worth exploring include: Do different age groups, religious backgrounds, or grief types require differentiated interfaces and commemorative modalities? Can algorithms be designed to support grief rather than standardize it? These questions touch not only on user experience optimization, but also on the moral transformation of death culture in the age of artificial intelligence. Ultimately, AI-based commemoration is not a neutral extension of human emotion, but a complex technological force that intervenes in subjectivity, ethical judgment, and cultural meaning.

## Data Availability

The original contributions presented in the study are included in the article/Supplementary Material, further inquiries can be directed to the corresponding authors.
